# Altered extracellular matrix and mechanotransduction gene expression in rat bone tissue following long-term estrogen deficiency

**DOI:** 10.1093/jbmrpl/ziae098

**Published:** 2024-07-24

**Authors:** Syeda Masooma Naqvi, Laura M O’Sullivan, Hollie Allison, Vincent J Casey, Jessica Schiavi-Tritz, Laoise M McNamara

**Affiliations:** Mechanobiology and Medical Devices Research Group (MMDRG), Biomedical Engineering, School of Engineering, College of Science and Engineering, University of Galway, Galway, H91 HX31, Ireland; Mechanobiology and Medical Devices Research Group (MMDRG), Biomedical Engineering, School of Engineering, College of Science and Engineering, University of Galway, Galway, H91 HX31, Ireland; Mechanobiology and Medical Devices Research Group (MMDRG), Biomedical Engineering, School of Engineering, College of Science and Engineering, University of Galway, Galway, H91 HX31, Ireland; Mechanobiology and Medical Devices Research Group (MMDRG), Biomedical Engineering, School of Engineering, College of Science and Engineering, University of Galway, Galway, H91 HX31, Ireland; Mechanobiology and Medical Devices Research Group (MMDRG), Biomedical Engineering, School of Engineering, College of Science and Engineering, University of Galway, Galway, H91 HX31, Ireland; University of Lorraine, CNRS, LRGP, F-54000 Nancy, France; Mechanobiology and Medical Devices Research Group (MMDRG), Biomedical Engineering, School of Engineering, College of Science and Engineering, University of Galway, Galway, H91 HX31, Ireland

**Keywords:** bone matrix, estrogens and SERMs, osteocytes, molecular pathways—remodeling, osteoporosis

## Abstract

Osteoporosis is primarily associated with bone loss, but changes in bone tissue matrix composition and osteocyte mechanotransduction have also been identified. However, the molecular mechanisms underlying these changes and their relation to bone loss are not fully understood. The objectives of this study were to (1) conduct comprehensive temporal gene expression analyses on cortical bone tissue from ovariectomized rats, with a specific focus on genes known to govern matrix degradation, matrix production, and mechanotransduction, and (2) correlate these findings with bone mass, trabecular and cortical microarchitecture, and mineral and matrix composition. Microarray data revealed 35 differentially expressed genes in the cortical bone tissue of the ovariectomized cohort. We report that catabolic gene expression abates after the initial accelerated bone loss period, which occurs within the first 4 wk of estrogen deficiency. However, in long-term estrogen deficiency, we report increased expression of genes associated with extracellular matrix deposition (Spp1, COL1A1, COL1A2, OCN) and mechanotransduction (Cx43) compared with age-matched controls and short-term estrogen deficiency. These changes coincided with increased heterogeneity of mineral-to-matrix ratio and collagen maturity, to which extracellular matrix markers COL1A1 and COL1A2 were positively correlated. Interestingly, mineral heterogeneity and collagen maturity, exhibited a negative correlation with PHEX and IFT88, associated with mechanosensory cilia formation and Hedgehog (Hh) signaling. This study provides the first insight into the underlying mechanisms governing secondary mineralization and heterogeneity of matrix composition of bone tissue in long-term estrogen deficiency. We propose that altered mechanobiological responses in long-term estrogen deficiency may play a role in these changes.

## Graphical Abstract

**Graphical Abstract:**
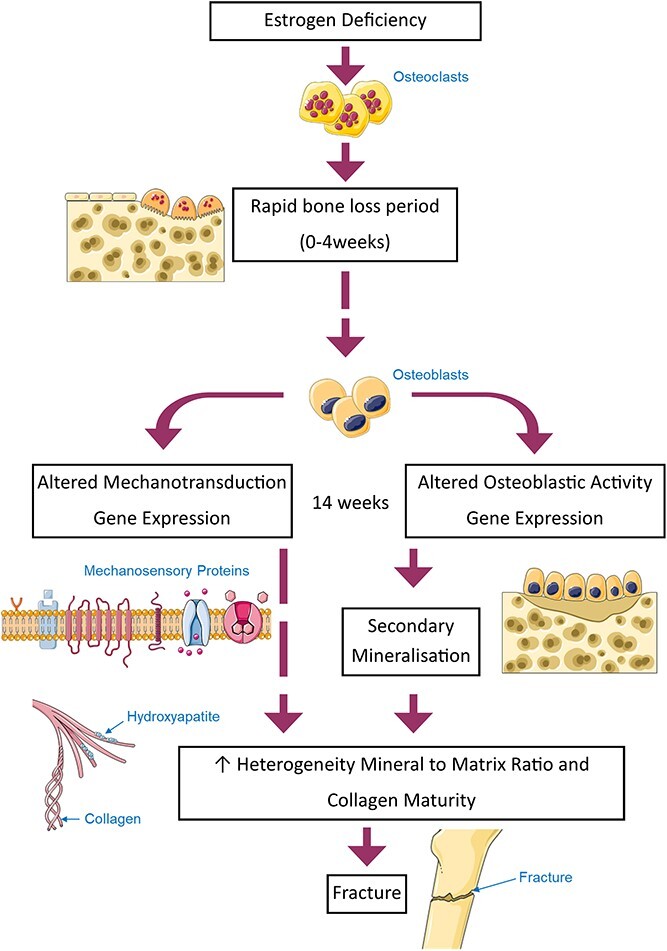

## Introduction

Osteoporosis is a debilitating bone disease, in which severe bone loss occurs leading to fractures of the hip, wrist, or vertebrae. Postmenopausal women experience significant bone loss, which is associated with a reduction in circulating estrogen at the onset of menopause and associated activation of osteoclast bone resorption. Therapies targeting bone-resorbing osteoclasts (bisphosphonates) have been the standard treatment for many years, albeit that they are only effective in preventing approximately half of associated bone fractures. Anabolic therapies, PTHrP and anti-sclerostin therapy, seek to prevent bone fracture by stimulating bone formation and decreasing bone resorption. The anti-sclerostin therapy has shown significant potential, but the anabolic effects are transient and bone formation dissipates within 2-9 mo.^1-5^ With an aging population, there is a significant and growing impact of osteoporosis on society, and it is projected that the global economic burden will reach $132 billion by 2050.^6^

Approximately 40% of women over 50 will suffer a fracture related to postmenopausal osteoporosis during their lifetime,^7^ which can lead to fracture nonunions, immobility, severe pain, and deformity, and an increase in mortality rates by 10%-20%.^3^ The disease is most commonly diagnosed by DXA, which quantifies BMD as a surrogate to predict the likelihood of fracture, albeit that bone loss cannot entirely predict fracture risk, particularly in younger patients.^8-12^ FRAX and Garvan fracture risk calculators incorporate BMD measurements and patient data to estimate fracture risk or include a second BMD measurement later in disease, but these approaches do not improve predictions of incident fractures.^11^^,^^12^ This is because bone strength and fracture susceptibility are also governed by bone tissue composition (mineral and collagen), microarchitectural characteristics, and the degree of tissue microdamage.^13-17^ These microstructural properties can increase fracture susceptibility despite seemingly normal bone density levels. Although many studies have demonstrated changes in tissue mechanical properties, mineral crystal size, collagen synthesis and cross-linking, and bone mineral and collagen heterogeneity,^18-25^ there are conflicting results regarding changes in mineral and collagen composition. These discrepancies may be attributed to variations in the duration of estrogen deficiency and the specific anatomical location from which the bone tissue is analyzed. Indeed, changes in bone matrix properties and tissue mineralization have been reported to arise secondary to bone loss, specifically occurring in longer term estrogen deficiency for rat and ovine models of osteoporosis.^18^^,^^26-28^ Most notably, this was demonstrated in a temporal in vivo study of bone loss and mineral distribution in ovariectomized rat tibiae in vivo.^28^ The ovariectomized rat model mirrors the bone loss and remodeling arising in humans following the onset of estrogen deficiency due to the menopause, albeit that bone loss is accelerated in the rat ovariectomy model relative to human osteoporosis. Interestingly, changes in the mechanical environment of osteocytes have also been reported during early-stage osteoporosis, but this environment was restored in the later stages of estrogen deficiency, which might be associated with secondary changes in tissue properties.^27^^,^^29^^,^^30^ We also determined temporal changes in the lacunar-canalicular network, which indicate that perilacunar remodeling occurs and alter mechanosensory capacity of the osteocyte network.^31^

In vitro, it has been reported that estrogen deficiency alters mechanosensory protein assembly (integrins, primary cilia) in osteocytes and osteoblasts and leads to changes in mechanotransduction and associated gene expression (RANKL, OPG, Cox2, Runx2, Spp1),^32-34^ as well as osteocyte regulation of osteoclast formation and matrix degradation.^35^ In a 3D ex vivo estrogen deficiency model, we reported an increase in osteocyte differentiation, mineralization markers (BSP, Spp1, Alpl, calcium), and pro-osteoclastogenic gene expression (RANKL/OPG ratio) in estrogen-deficient and mechanically stimulated cells.^36^ Altogether, these studies provide evidence that, in addition to bone loss, osteoporosis involves temporal alterations in bone tissue composition and mechanotransduction. However, the molecular mechanisms underlying these changes and the time sequence in which they occur are not fully understood.

Gene expression patterns can provide an insight into the underlying molecular mechanisms contributing to secondary mineralization and the pathogenesis of osteoporosis. Rapid and dynamic gene expression changes have been reported at the onset of estrogen deficiency. In an ovariectomized mouse model, an increase in expression of genes associated with osteoclastogenesis (e.g., RANKL, Mmp9) were identified by 2 weeks (wk) after ovariectomy, whereas those known to govern osteoblast differentiation and matrix production (e.g., Runx2, COL1A1) were decreased.^37^ Microarray analyses have identified candidate genes and pathways associated with osteoporosis.^38-44^ Analysis of gene expression profiles in the bone marrow of mice revealed differentially expressed genes associated with immune system function, enzymatic processes, and cellular development (IL7R, DPP4, BLNK, CD79A, CD72, CAPSL, CPM, GPX3, CD80).^38^^,^^39^ Studies of microarray datasets from human osteoporotic bone have identified variations in genes associated with osteogenic differentiation, metabolism, Mapk and calcium signaling, cGMP-PKG, cell adhesion (Rap1), and endocytosis.^40^^,^^41^ A meta-analysis study of data from six microarray studies of human female bone tissue identified alterations in gene expression associated with osteoclast differentiation and various signaling pathways (B cell receptor, MAPK, chemokine, insulin).^42^ Osteoporotic osteoblasts cultured in vitro have shown differential expression of genes involved in bone formation, mineralization, and cell signaling (PTN, CXCL2, COL15Α1, IBSP, AOX1, MT1G, GSR, AND TXNRD1).^43^ A comparative transcriptome analysis of osteoblasts obtained from individuals with osteoporosis identified changes in gene expression known to govern chondrocyte development, bone mineralization, BMP and Wnt signaling, and DNA methylation.^44^ These studies contribute to our understanding of the molecular mechanisms underlying osteoporosis. However, to date, such studies have been conducted at a single time-point, whereas it is becoming increasingly clear that changes in bone loss, tissue composition, and mechanosensation are time-dependent. Therefore, temporal analysis of gene expression is required to provide a comprehensive understanding of the sequence of changes in bone loss and secondary mineralization.

The first objective of this study was to conduct comprehensive temporal microarray analyses of gene expression in cortical bone from the tibiae of ovariectomized rats, with a specific focus on genes that govern bone matrix production, resorption, and mechanotransduction. The second objective was to correlate these gene expression profiles to temporal variations of trabecular and cortical microarchitecture and mineral and organic matrix composition, as determined by high-resolution micro-computed tomography (micro-CT) and Raman spectroscopy analyses of trabecular tissue from the proximal tibia metaphysis from an OVX rat model.

## Materials and methods

### Animal model

Animal procedures were conducted under licence from the Animal Care and Research Ethics Committee of University of Galway and the national Health Products Regulatory Authority. Female retired breeder Wistar rats (6-month-old, Charles River Ireland) that underwent at least one pregnancy/lactation cycle were randomly assigned to groups for either (1) bilateral ovariectomy (OVX, *n* = 10) or (2) a sham operation (SHAM, *n* = 10) (Body weight: OVX: 410 ± 68 g, SHAM: 445 ± 17 g). Success of ovariectomy was confirmed in necropsy by the absence of ovaries and atrophy of the uterine horns. The rats were pair-housed in controlled conditions: temperature (approximately 22 °C), humidity (around 50%-60%), and a 12-h light–dark cycle. Their housing provided clean bedding of wood shavings and nesting material, with *ad libitum* access to food and water. A specialized laboratory diet, free from contaminants affecting study outcomes and in pellet form, was their sole nutrition source throughout the study period. The rats were sacrificed at (1) 4 wk postovariectomy (short-term estrogen deficiency, OVX, *n* = 5, SHAM, *n* = 5) or (2) 14 wk post-ovariectomy (long-term estrogen deficiency, OVX, *n* = 5, SHAM, *n* = 5). A power analysis was conducted to determine the sample size for detecting longitudinal differences in trabecular thickness and bone volume fraction using in vivo micro-CT in ovariectomized rats compared with controls. The effect size was estimated based on previous measurements of trabecular thickness and bone volume fraction in the same animal model^45^ using G^*^Power software (Universitat Kiel, Germany) to achieve a statistical power of 80%, which established that a group size of 5 was necessary. The selection of time points was informed by previous longitudinal micro-CT studies in OVX rat models. These studies demonstrated a rapid decrease in bone volume and microarchitecture within the first 4 wk post-OVX, followed by a slower rate of bone loss over the long term (14 wk).^28^ Notably, during this early period of estrogen deficiency, no significant alterations in mineral distribution have been reported, whereas in the later stage of estrogen deficiency, the tissue mineral composition is increased.^28^ Thus, the choice of time points included both the initial acute bone loss response and the subsequent adaptations in bone structure following estrogen deficiency, providing valuable insights into the dynamics of bone remodeling in this context.

### High-resolution micro-CT

Micro-CT scanning quantified changes in trabecular and cortical microarchitecture and BMD distribution in ovariectomized rat bone tissue. Tibiae were harvested and stored in phosphate-buffered saline (PBS) soaked gauze at −20 °C. Scans were performed using a high-resolution micro-CT system (μCT100, Scanco Medical AG, Basserdorf, Switzerland) with an isotropic resolution of 3 μm. The following scan settings were used: X-ray tube potential of 70 kV, current 50 μA, integration time 1500 ms, 1500 projections per 180°, and a 0.5-mm-thick aluminum filter was used to reduce beam hardening artifacts.

The region of interest (ROI) was a 1.5-mm section of metaphyseal bone located 1 mm below the growth plate in the proximal tibia. ImageJ and Scanco software were used for quantification of bone volume fraction (BV/TV). BMD distribution analysis was conducted to provide a quantitative description of the mineral composition of bone from the proximal tibia. Mean tissue mineral density (Mean Mineral Density, mg HA/ccm) and the full width at half maximum of the mineral distribution curve (Mineral Heterogeneity, mg HA/ccm) were analyzed for all groups.

At baseline (day 0), the right tibia of each animal from the SHAM and OVX animal cohort was scanned with an isotropic resolution of 15 μm, using an in vivo micro-CT system (VivaCT40, Scanco Medical AG, Basserdorf Switzerland). Scan parameters included a 70 kVp X-ray tube potential, 114 μA current, 300 ms integration time, 1000 projections per 180°, and a 0.5-mm-thick aluminum filter to reduce artifacts. Animals were anesthetized with isoflurane, and respiratory rates were monitored. The limb was positioned and fixed for scanning, lasting about 20 minutes (min), and emitting ~850 mGy. An ROI was chosen, 3 mm below the growth plate in the proximal tibia, using semi-automated segmentation. A small ROI was chosen for micro-CT, rather than scanning the entire metaphyseal volume, to minimize the scanning duration to ~20 min to prevent a drop in animal body temperature. A global threshold value of 614 mg HA/ccm was applied to separate bone from other components.

### Raman spectroscopy and spectral analysis

Raman spectroscopy and spectral analysis was conducted to quantify changes in mineral and matrix composition in ovariectomized rat bone tissue by assessing the mineral-to-matrix ratio and collagen maturity and estimating the spatial heterogeneity of these parameters. A 3-mm section of metaphyseal trabecular bone was cut from the metaphyseal region of the proximal tibia using a diamond blade on a low-speed sectioning saw (ISOMET™ Low Speed Saw, Buehler, IL, USA), such that the trabecular structure was exposed. Ultrasonic washing in dH_2_O and short bouts of centrifuging (1000 rpm) extracted marrow from within the spaces of the trabecular structure, to facilitate epoxy embedding later. The sections were put through a series of 5-min ascending ethanol washes for dehydration and then embedded in epoxy resin (EpoThin2TM, Buehler, IL, USA) under vacuum for 2 min to support the strut-like network of trabeculae. The test surface was prepared with a diamond blade (ISOMETTM Low-Speed Saw, Buehler, IL, USA) and polished using a series of diamond suspension pastes (9, 3, 1.5, and 0.05 μm) with polishing cloths on a semi-automated polisher (MetaServ® 250 Grinder-Polisher with Vector® LC Power Head, Buehler, IL, USA). The samples were washed in dH_2_O using an ultrasonic bath (VWR, Dublin, Ireland) between each polishing step. We analyzed metaphyseal trabeculae from specific regions in the proximal tibia to ensure consistency and comparability. In each region of the sample, one transversely oriented trabecula was identified for Raman spectroscopy analysis (three trabeculae from each animal). All spectra of Raman shifts were obtained with a LabRAM HR 800 Confocal Raman microscope (HORIBA Jobin Yvon Ltd., Middlesex, UK). The instrument was operated at 785 nm with a spectral resolution of 1 cm^−1^. A spectral map (with dimensions 48 μm × 96 μm, 8 μm step size) containing 72 data points was obtained across the width of one trabecula (Figure S1). Raman spectra were baseline corrected (third degree polynomial) to account for fluorescence, normalized relative to spectra from the same map, and smoothed using a Savitzky–Golay filter (fourth degree polynomial), and Gaussian curve fitting was performed using LapSpec 5 software (HORIBA Jobin Yvon Ltd., Middlesex, UK). The following parameters were calculated for each spectrum using a custom-written Python script (1) mineral-to-matrix ratio, expressed as the integrated area ratio of the v_1_PO_4_^−3^ phosphate band (959 cm^−1^) to amide I band (1660 cm^−1^),^46^ and (2) collagen maturity, based on the relative Pyridinoline content, was expressed as the ratio of Raman intensity of the amide I band (1660 cm^−1^) to the area of the amide I band (1620-1700 cm^−1^).^47-50^ The spatial heterogeneity of each parameter was estimated using the FWHM of the Gaussian curve fitted to the pixel histograms for each Raman map using a custom-written MATLAB script.

### Nanoindentation

Nanoindentation was performed on trabecular tissue from the proximal tibia metaphysis. The surface was prepared using a diamond blade on a low-speed saw (ISOMETTM Low-Speed Saw, Buehler, IL, USA) and polished with diamond suspension pastes (9, 3, 1.5, and 0.05 μm) on a semi-automated polisher (MetaServ® 250 Grinder-Polisher with Vector® LC Power Head, Buehler, IL, USA). Nanoindentation testing employed a NanoIndenter G200 (Keysight Technologies, CA, USA) with a Berkovich diamond indenter, ensuring environmental isolation with tests conducted on a vibration isolation table within a thermal/sound-insulated cabinet. A transversely oriented trabecula was identified for nanoindentation (three trabeculae per animal). An array of 12 indents (2 parallel rows of 6) was made across each trabecula, with indents at least 5 μm from the edge and 15 μm from neighboring indents. The loading profile included two conditioning steps (5 and 10 mN) before reaching a maximum load of 20 mN, each held for 120 s, then unloading to 10% of the load over 90 s. Post-unloading, the indenter was held at 10% of the peak load for 120 s to correct for thermal drift. The average maximum indentation depth was 1.5 μm. Load–displacement curves were generated for each indent, and the Oliver and Pharr method was used to estimate the elastic modulus from the upper 25% of the final unloading segment.

### Microarray

To investigate the molecular changes associated with estrogen deficiency-induced bone loss, a microarray approach was implemented to facilitate temporal analyses of gene expression associated with bone matrix production, resorption, and mechanotransduction. The metaphyseal region of the proximal tibia was dissected, washed with DNase/RNase-free PBS, and cortical bone was isolated. To isolate cortical bone for RNA extraction, we scraped the bone to eliminate any endosteum and then washed it multiple times in DNase water to ensure the removal of any bone marrow residue. RNA extraction was conducted only on the cortical bone in OVX rats because of the diminishing trabecular bone volume over time post-ovariectomy, wherein by week 14, there was minimal trabecular tissue remaining, posing challenges in obtaining ample quantities of high-quality RNA. The samples were snap frozen in liquid nitrogen and stored at −80 °C for microarray analysis. Total RNA was isolated (RNeasy Fibrous Tissue Mini Kit, Qiagen) and cDNA was obtained using a SuperScript VILO cDNA synthesis kit (Invitrogen). A customized microarray (Applied Biosciences) was designed to study (1) bone anabolism and extracellular matrix genes (Spp1, OPG, COL1A1, COL1A2, OCN, LRP5, Alpl, Tgfβ1, Lox, Ager, DMP1, PHEX, Klf10, Fn1), (2) bone catabolism and extracellular matrix degradation genes (RANKL, RANK, Nfatc-1, Mmp9, Mmp13, Sost, CtsK, Wnt10B, CCR2, IL1B, TP53, Casp3, Lamp1, ATG7, Rptor), and (3) mechanotransduction and mechanosensation genes (Vcl, Itga5, Itgav, Itgb1, Cx43, Esr1, Axin2, IFT88, TRPV4, Adcy6, Ryr, Pkd1). The selection and categorization of genes were carried out based on specific research objectives and the nature of the custom microarray (Applied Biosciences) used in the study. The rationale for categorizing the genes in this manner was to align them with the primary biological processes they are associated with. Although these categories may not encompass the full complexity of gene functions, the categorization aimed to facilitate the analysis and interpretation of data related to bone health, remodeling, and mechanosensitivity within the context of the study’s objectives. Independent batches were used to run a total of 20 microarrays, with 5 microarrays each for Sham and OVX groups at 4 and 14 wk, respectively. Hybridization was performed using a TaqMan® Fast Advanced Master Mix and the microarray was performed using a StepOnePlus™ Real-Time PCR System, with analysis by the Pfaffl method using Rpl13a as the reference gene. Results are expressed as relative quantitative changes to samples from age-matched SHAM controls.

### Two-component principal component analysis

Two-component principal component analysis (PCA) was performed using R Statistical Software (R Studio 2022.02.3) to determine the relationship between genes within the dataset for 4- and 14-wk groups for SHAM and OVX animals. The ellipses around week 4 SHAM (continuous blue line), week 14 SHAM (broken blue line), week 4 OVX (continuous orange line), and week 14 OVX (broken orange line) define the region that contains 95% of all samples. Principal component 1 and 2 percentages represent variance captured by each principal component. The direction the vectors point to show that the gene expression is greater for that ellipse. Vectors also reveal gene correlation: small angle implies positive correlation, a large angle suggests negative correlation, and a 90° angle indicates no correlation between two genes.

### Correlation analysis

R Statistical Software (R Studio version 2022.02.3) was utilized to construct correlation matrices, enabling the examination of relationships between ex vivo measurements and gene expression data. These matrices visually depict correlation strength through color gradients: dark blue indicating negative correlation, dark red indicating positive correlation, and white representing no correlation. Additionally, the software generated corresponding numerical values (*r*-values and *p*-values) quantifying the strength and significance of these correlations. The correlation coefficient (*r*-value) measures the strength and direction of a linear relationship between two variables. A positive value indicates a positive correlation (as one variable increases, the other tends to increase), while a negative value signifies a negative correlation (as one variable increases, the other tends to decrease). The *p*-values associated with each correlation indicate the statistical significance of the correlation. In this context, a lower *p*-value (usually below a certain threshold such as 0.05 or 0.01) suggests that the observed correlation is unlikely to have occurred by chance and is more likely a meaningful relationship. This comprehensive output offers both visual and statistical insights into the associations observed within the datasets.

### Statistics

Statistical analyses were performed using GraphPad Prism (version 8) software. One-way ANOVA (Kruskal-Wallis) was used to analyze the statistical significance of the differences between the OVX and age-matched control (SHAM) groups and between time points, for all parameters of interest. The results are displayed as mean ± SD. Significance was accepted at a level of *p*≤ 0.05.

## Results

We conducted a microarray analysis of gene expression data obtained from cortical bone tissue samples collected from OVX and SHAM animals at 4 and 14 wk. Our findings indicate differential expression of 35 genes in the ovariectomized cohort at 14 wk.

### Increased bone loss within the first 4 wk of ovariectomy, at which time bone catabolism and extracellular matrix degradation gene expression abate, whereas bone loss plateaus by 14 wk

**Figure 1:**
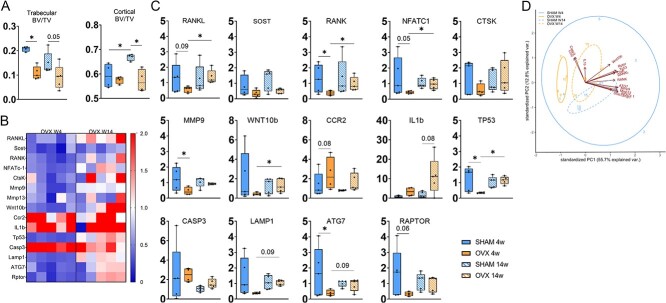
Bone catabolism and extracellular matrix degradation gene expression analysis of bone tissue at 4 and 14 wk. (A) Analyses of tissue microarchitecture and composition by high-resolution micro-CT scanning of the proximal tibia from SHAM and OVX animals. (B) Heat map reflecting gene expression for OVX animals, with fold value of expression normalized to SHAM animals at respective time points; negative values indicate downregulation (fold change < 1), neutral values indicate no change (fold change = 1), and positive values indicate upregulation (fold change > 1). (C) Gene expression (RANKL, Sost, RANK, NFatc1, CtsK, Mmp9, Wnt10B, CCR2, IL1B, TP53, Casp3, Lamp1, ATG7, Rptor). (D) Two-component PCA of gene expression data identifies differential patterns between OVX time points. ^*^p≤.05; ^*^^*^^*^p≤.001.

To determine bone loss and changes in bone microarchitecture during estrogen deficiency, high-resolution micro-CT was conducted on the proximal tibia from OVX and age-matched control rats (Figure 1A, Table S1). Micro-CT analysis of rats sacrificed 4 wk after surgery confirmed that trabecular bone volume fraction was significantly reduced in OVX animals compared with age-matched controls. Importantly, at 14 wk postsurgery, OVX animals showed a comparable trabecular bone volume fraction to age-matched controls showing a plateau in bone loss. Micro-CT analysis of rats sacrificed 14 wk after surgery confirmed that cortical bone volume fraction was significantly reduced in OVX animals compared with age-matched controls (Figure 1A).

The heatmap illustrates the expression patterns of genes related to bone catabolism and extracellular matrix degradation, showing a downregulation (in blue) in the gene expression of specific markers in the 4 wk OVX group, while the expression is more variable in the 14 wk OVX groups (Figure 1B). Statistical comparisons confirmed a significant decrease in gene expression of RANK, TP53, and ATG7 in 4 wk OVX relative to 4 wk SHAM. Additionally, there was a significant increase in gene expression of RANKL, RANK, NFATc-1, Wnt10B, and TP53 in 14 wk OVX relative to 4 wk OVX (Figure 1C).

We conducted a deeper exploration of the gene expression data, utilizing a PCA with a data matrix comprising 20 samples and 300 features. PCA identified distinct clusters in the data, separated based on scores on the first principal component (Figure 1D), corresponding to 4 and 14 wk OVX, confirming an overall differential gene expression between the two time points. PC1 and PC2 together accounted for 68.5% of the total variance, which means that additional components may be associated with multivariate patterns. However, since a multivariate pattern already emerged based on PC1 and PC2, we did not further evaluate the remaining components. The variance between the two OVX time points is explained by markers RANKL, RANK, NFATc-1, Mmp9, Wnt10β, TP53, Lamp1, ATG7, and Rptor.

### Upregulation in extracellular matrix gene expression in bone tissue from ovariectomized animals, coinciding with increased mineral and matrix heterogeneity and heterogeneity of collagen maturity

**Figure 2:**
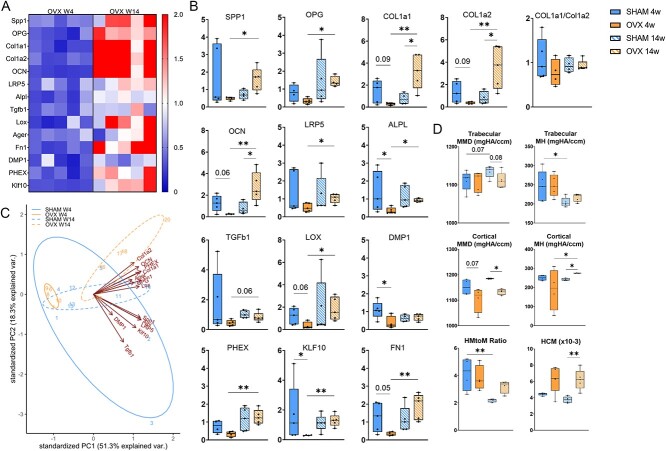
Bone anabolism and extracellular matrix production gene expression analysis of bone tissue at 4 and 14 wk. (A) Heat map reflecting gene expression for OVX animals, with fold value of expression normalized to SHAM animals at respective time points; negative values indicate downregulation (fold change < 1), neutral values indicate no change (fold change = 1), and positive values indicate upregulation (fold change > 1). (B) Gene expression (Spp1, OPG, COL1A1, COL1A2, OCN, LRP5, Alpl, Tgfβ1, lox, DMP1, PHEX, Klf10, Fn1). (C) Two-component PCA gene expression data reveals differential expression patterns between OVX time points and SHAM controls. (D) Analyses of tissue microarchitecture and composition by high-resolution micro-CT scanning and Raman spectroscopy of the proximal tibia from SHAM and OVX animals. ^*^p≤.05; ^*^^*^p≤.01; ^*^^*^^*^p≤.001.

The heatmap visualizes gene expression patterns related to bone anabolism and the extracellular matrix, revealing a downregulation (in blue) in the gene expression of all markers in the 4 wk OVX group. Conversely, an upregulation (in red) is observed in the gene expression of specific markers, namely Spp1, COL1A1, COL1A2, and OCN, in the 14 wk OVX groups, when compared with the SHAM group (Figure 2A). Statistical comparisons reveal a significant downregulation of genes linked to bone anabolism and extracellular matrix production at OVX wk 4, when compared with age-matched SHAM controls, with DMP1 and ALP exhibiting a 3-fold decrease (Figure 2B). Interestingly, at wk 14, genes associated with bone anabolism and extracellular matrix production, including COL1A1 (3-fold), COL1A2 (3-fold), and OCN (2-fold), were significantly upregulated in comparison to their age-matched SHAM controls (Figure 2B). Statistical comparisons further revealed an upregulation in Spp1, OPG, COL1A1, COL1A2, OCN, LRP5, Alpl, Lox, PHEX, Klf10, and Fn1 genes in 14 wk OVX relative to 4 wk OVX (Figure 2B). There was no significant difference in Col1A1/Col1A2 in OVX animals in comparison to age-matched SHAM controls for both time points (Figure 2B).

We conducted a deeper exploration of the gene expression data, utilizing a PCA analysis with a data matrix comprising 20 samples and 280 features. PCA revealed two distinct clusters corresponding to 4 and 14 wk OVX (Figure 2C). The variance between the two OVX time points is explained by markers Spp1, OPG, COL1A1, COL1A2, OCN, LRP5, Alpl, Tgfβ1, Lox, PHEX, Klf10, and Fn1.

High-resolution micro-CT scans of the proximal tibia from estrogen-deficient (OVX) and age-matched control rats were performed to assess mineralization changes. Analysis of rats sacrificed 14 wk postsurgery revealed similar trabecular mean mineral densities and mineral heterogeneity compared with the control group (Figure 2D, Table S1). There was a significant reduction in cortical mean mineral density and a significant increase in cortical mineral heterogeneity in animals 14 wk postovariectomy compared with age-matched SHAM control group (Figure 2D, Table S1). To determine the changes in bone matrix composition during estrogen deficiency, Raman spectroscopy was conducted on individual trabeculae of the tibia from OVX and age-matched control rats, which were sacrificed at 4 or 14 wk after surgery (Figure 2D, Table S2). Four wk into estrogen deficiency, SHAM and OVX trabecular tissue exhibited similar heterogeneity of mineral-to-matrix ratio and collagen maturity. By 14 wk of estrogen deficiency, heterogeneity of mineral-to-matrix ratio was significantly increased in the OVX group compared with controls. Similarly, OVX tissue had significantly increased heterogeneity of collagen maturity compared with SHAM tissue at wk 14 postsurgery.

Nanoindentation was performed on trabecular tissue from the proximal tibia metaphysis (of these same animals) to determine tissue-level mechanical properties (see Figure S2). There was a nonsignificant (*p*=.07) decrease in elastic modulus in OVX animals 14 wk postovariectomy compared with age-matched SHAM group.

### Altered expression of genes associated with mechanotransduction and mechanosensation in ovariectomized bone tissue

**Figure 3:**
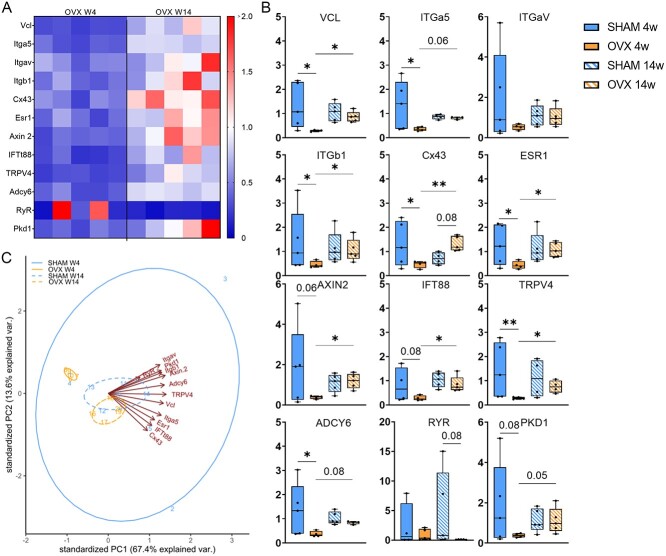
Mechanotransduction and mechanosensation gene expression analysis of cortical bone tissue in ovariectomized animals at 4 and 14 wk. (A) Heat map reflecting gene expression for OVX animals, with fold value of expression normalized to SHAM animals at respective time points; negative values indicate downregulation (fold change < 1), neutral values indicate no change (fold change = 1), and positive values indicate upregulation (fold change > 1). (B) Mechanotransduction/sensation gene expression (Vcl, Itga5, Itgav, Itgb1, Cx43, Esr1, Axin2, IFT88, TRPV4, Adcy6, Ryr, Pkd1). (C) Two-component PCA of gene expression data reveals differential expression patterns between OVX time points PCA of microarray dataset. ^*^p≤.05.

The heatmap illustrates the expression patterns of genes related to mechanotransduction and mechanosensation, showing a downregulation (in blue) in the gene expression of specific markers in the 4 wk OVX group, while the expression appears to be more variable in the 14 wk OVX groups (Figure 3A). At wk 4, statistical comparisons reveal a significant downregulation in Vcl, Itga5, Itgb1, Cx43, Esr1, TRPV4, and Adcy6 in OVX bone compared with age-matched SHAM controls (Figure 3B). At wk 14, a notable upregulation of Cx43 was detected, 1.5-fold increase in OVX bone in comparison to age-matched SHAM controls (Figure 3B). Statistical comparisons further revealed an upregulation in Vcl, Itgb1, Cx43, Esr1, Axin2, IFT88, and TRPV4 in 14 wk OVX relative to 4 wk OVX (Figure 3B).

We conducted a deeper exploration of the gene expression data, utilizing a PCA with a data matrix comprising 20 samples and 240 features. PCA revealed two distinct clusters corresponding to 4 wk OVX and 14 wk OVX (Figure 3C). The variance between the two OVX time points is explained by markers Vcl, Itga5, Itgav, Itgb1, Cx43, Esr1, Axin2, IFT88, TRPV4, Adcy6, and Pkd1.

### Correlation matrix revealed important correlations between microarchitecture, mineral and matrix composition, and genes associated with matrix production and mechanotransduction

Correlation analysis was first conducted on the complete data set, encompassing both time points and the experimental groups (Figures S3 and S4, Tables 1 and 2). This comprehensive analysis offers an overall perspective on variable relationships across time. Before exploring correlations between microarchitecture, mineral-matrix composition, and gene expression data, internal relationships within each dataset were examined to validate established correlations. In microarchitecture and mineral-matrix composition data (Table S3), trabecular bone volume fraction and number showed significant negative correlations with heterogeneity of collagen maturity. Trabecular number and mineral heterogeneity showed significant positive correlations with mineral-matrix ratio, its heterogeneity and collagen maturity. In gene expression data (Table S4), bone anabolism and extracellular matrix genes and bone catabolism and extracellular matrix degradation genes showed significant positive correlations with several mechanotransduction and mechanosensation markers. These correlations confirm established correlations and substantiate the associative results herewith.

**Table 1:** Correlation of trabecular ex vivo data to gene expression data for the complete dataset (weeks 4 and 14 groups).

Gene	BV/TV	TN	MMD	MH	HMtoMR	CM	HCM
**OPG**	ns	ns	ns	*r* = −0.53; *p*=.02	ns	*r* = −0.55; *p*=.01	ns
**Ager**	ns	ns	ns	*r* = −0.46; *p*=.04	ns	*r* = −0.48; *p*=.03	ns
**PHEX**	ns	ns	ns	*r* = −0.61; *p*=.004	*r* = −0.47; *p*=.04	*r* = −0.65; *p*=.002	ns
**DMP1**	*r* = 0.50; *p*=.02	*r* = 0.48; *p*=.03	ns	ns	ns	ns	ns
**Sost**	ns	ns	*r* = 0.54; *p*=.01	ns	ns	ns	*r* = −0.57; *p*=.008
**CCR2**	ns	ns	ns	ns	ns	ns	*r* = 0.44; *p*=.05
**IL1B**	*r* = −0.55; *p*=.01	*r* = −0.50; *p*=.02	ns	ns	ns	ns	*r* = 0.48; *p*=.03
**Mmp9**	ns	ns	ns	ns	ns	ns	*r* = −0.45; *p*=.04
**Itga5**	*r* = 0.48; *p*=.03	ns	ns	ns	ns	ns	ns
**Adcy6**	*r* = 0.46; *p*=.04	ns	ns	ns	ns	ns	*r* = −0.45; *p*=.05
**IFT88**	ns	ns	ns	*r* = −0.49; *p*=.03	ns	*r* = −0.55; *p*=.01	ns
**Vcl**	*r* = 0.46; *p=*.04	ns	ns	ns	ns	ns	*r* = −0.48; *p*=.03
**TRPV4**	*r* = 0.48; *p*=.03	ns	ns	ns	ns	ns	*r* = −0.48; *p*=.03

ns = not significant.

*r* = correlation magnitude.

*p* = *p-*value.

**Table 2:** Correlation of cortical microCT data to gene expression data for the complete dataset (weeks 4 and 14 groups).

**Gene**	**BV/TV**	**MMD**	**MH**
**Fn1**	ns	ns	*r* = 0.44; *p*=.04
**Alpl**	*r* = 0.44; *p*=.04	ns	ns
**Sost**	*r* = 0.61; *p*=.003	ns; *p*=.08	ns
**Ccr2**	ns	*r* = −0.55; *p*=.01	ns
**Mmp9**	*r* = 0.50; *p*=.02	ns	ns
**IFTt88**	*r* = −0.55; *p*=.01	ns; *p*=.07	ns; *p*=.07
**Vcl**	*r* = −0.47; *p*=.03	ns; *p*=.07	ns

ns = not significant.

*r* = correlation magnitude.

*p* = *p-*value.

There was a negative correlation between BV/TV and trabecular number with IL1B gene expression (*r* = −0.55 and −0.50, respectively), while a positive correlation was found between BV/TV and trabecular number with DMP1 (*r* = 0.50 and 0.48, respectively), see Figure S3 and Table 1. Mean mineral density was positively correlated with Sost expression (*r* = 0.54). Mineral heterogeneity and collagen maturity were negatively correlated with OPG (*r* = −0.53 and −0.55, respectively), Ager (*r* = −0.46 and 0.48, respectively), osteocyte marker PHEX (*r* = −0.61 and −0.65, respectively), and mechanotransduction marker IFT88 (*r* = −0.49 and −0.55, respectively). BV/TV was positively correlated with ALP (*r* = 0.44), Sost (*r* = 0.61), and MMP9 (*r* = 0.50), while a negative correlation was observed between BV/TV and mechanotransduction markers IFT88 and Vcl (*r* = −0.55 and −0.47, respectively), see Figure S4 and Table 2. Mean mineral density was negatively correlated with Ccr2 expression (*r* = −0.55). A positive correlation was observed between Mineral Heterogeneity and Fn1 gene expression (*r* = 0.44).

While analyzing correlations across the complete dataset offers a broad view of variable relationships over time, we conducted additional analyses at each time point to understand temporal patterns and changes. Specifically, we conducted additional correlation analyses at each individual time point of 4 wk (Figure S5, Table S5) and 14 wk (Figure S6, Table S6).

By 4 wk, bone anabolism and extracellular matrix markers Fn1, OCN, and Lox were positively correlated with bone volume fraction, and Spp1, Klf10, COL1A1, LRP5, Alpl, Tgfb1, and Lox were positively correlated with trabecular number (Figure S5, Table S5). Pro-apoptotic marker, TP53, was positively correlated with bone volume fraction. Bone catabolism markers RANKL, Sost, Rptor, TP53, NFATc1, Lamp1, ATG7, Mmp9, and Mmp13 were positively correlated with trabecular number. RANKL and Wnt10β were positively correlated with mineral heterogeneity. IL1b was negatively correlated with bone volume fraction and CCR2 was negatively correlated with trabecular number. Mechanotransduction markers Itga5, Itgb1, Pkd1, Axin2, Adcy6, RyR, Vcl, and TRPV4 were positively correlated to trabecular number. Markers Itgav, Pkd1, and Axin2 were positively correlated to mineral heterogeneity.

At 14 wk, extracellular matrix markers COL1A1 and COL1A2 were positively correlated with heterogeneity of collagen maturity (Figure S6, Table S6). Bone metabolism marker, OPG, was negatively correlated with collagen maturity. Apoptosis marker casp3 was negatively correlated with bone volume fraction. Inflammatory marker, CCR2, was positively correlated with heterogeneity of mineral to matrix ratio (Table S6). Mechanotransduction marker Cx43 was negatively correlated with mean mineral density.

## Discussion

**Figure 4:**
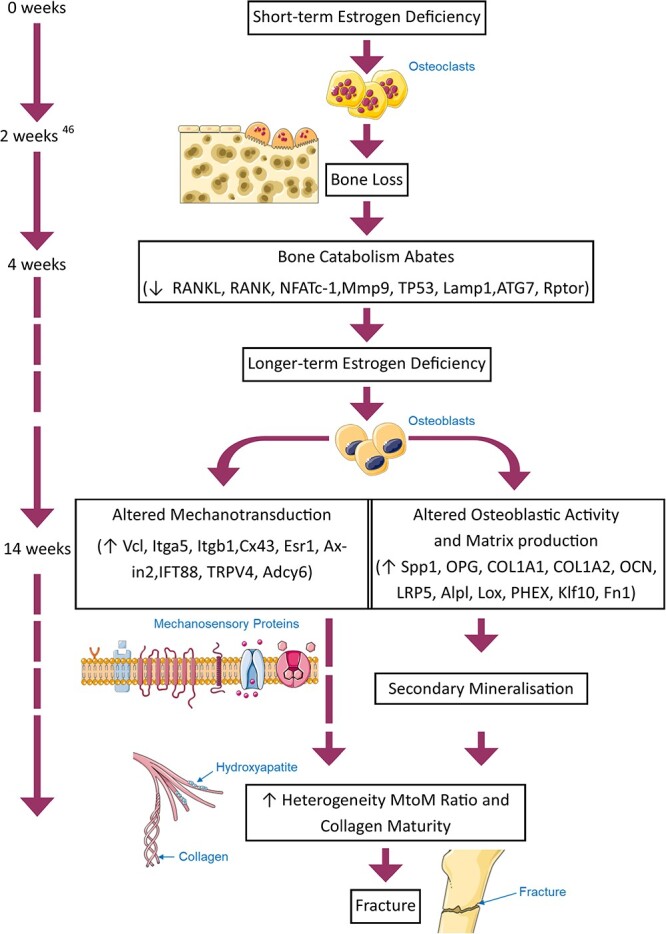
Flowchart depicting the research from this study in the context of a proposed theory of the sequence of events during long-term estrogen deficiency, which results in increased fracture risk.

This study reveals the temporal nature of changes in gene expression in bone tissue from ovariectomized animals. Specifically, we report that after the initial accelerated bone loss period, catabolic gene expression abates with a significant downregulation in RANK, TP53, and ATG7 in 4 wk OVX relative to 4 wk SHAM. However, in long-term estrogen deficiency, we report increased expression of genes associated with extracellular matrix deposition (COL1A1, COL1A2, OCN) (Figure 4). Temporally, a significant upregulation in ECM encoding gene expression was shown to arise from 4 to 14 wk in ovariectomized bone tissue (Spp1, OPG, COL1A1, COL1A2, OCN, LRP5, ALPL, LOX, Phex, KLF10, and FN1). Increased expression of genes associated with mechanotransduction and mechanosensation (Connexin 43 (Cx43)) relative to age-matched controls was also reported. Gene expression associated with mechanotransduction and mechanosensation by integrins and primary cilia (VCL, ITGb1, Cx43, AXIN2, IFT88, TRPV4) was also upregulated in OVX bone tissue from 4 to 14 wk. Interestingly, mineral heterogeneity negatively correlated with PHEX and IFT88, involved in mechanosensory cilia formation. It is important to note that tissue mineral heterogeneity is associated with increased fracture risk.^24^ Thus, we propose that altered mechanobiological responses in long-term estrogen deficiency may play a role in these changes. We provide the first insight into the underlying mechanisms governing secondary mineralization and heterogeneity of matrix composition of bone tissue in long-term estrogen deficiency.

There are some limitations that need to be considered in this study. First, although the ovariectomized rat is a well-established model for postmenopausal osteoporosis, the bone loss processes and metabolic rates are accelerated in the rat ovariectomy model relative to human osteoporosis, and rats have less mature, but functional, Haversian systems. However, the ovariectomized rat experiences an initial period of bone loss in estrogen deficiency followed by a return to a more balanced remodeling process as bone loss subsides.^28^^,^^45^^,^^51^ Similarly, humans experience rapid bone loss within the first 2 yr postmenopause, which then decelerates to ~1% per year.^52^ Thus, the insights gained from the ovariectomized rat model may not directly translate temporally to human postmenopausal osteoporosis. Second, the microarchitecture deterioration in OVX animals did not reflect the severity of bone loss and trabecular thickening previously reported in the proximal tibial metaphysis of ovariectomized rats after similar periods of estrogen deficiency.^25^^,^^53^ In vivo micro-CT scans indicated baseline differences in these cohorts (day 0), whereby trabecular number was significantly higher, and mineralization was significantly lower in OVX animals compared with SHAM animals (see Figure S6). These baseline differences were not explained by presurgery body weight but may relate to the number of breeding cycles, which may alter trabecular structure.^54^ Nonetheless, OVX animals experienced significant bone loss in the first 4 wk, and bone loss subsided thereafter, similar to published results of estrogen deficient rats,^28^ and the changes in mineral composition were detected albeit these were less marked. Third, we acknowledge the dependency of the peaks used in this study for the mineral-to-matrix ratio (959 and 1660 cm^−1^) on tissue organization and orientation.^46^ However, these are commonly analyzed due to their strong correlation with the mineral content and the organic matrix, respectively. Furthermore, we implemented consistent sample preparation and control measures to minimize the effects of tissue organization and orientation. Fourth, the microarray analysis was performed on cortical bone, due to insufficient trabecular bone volume in OVX animals to obtain adequate RNA samples, whereas the mineral/matrix composition analyses were conducted on trabecular bone. It is important to note that our study employed 6-mo-old retired breeder rats that underwent ovariectomy and were sacrificed after 14 wk, and so the rats experienced pronounced trabecular bone loss. Cortical bone loss is gradual in osteoporosis, whereas trabecular bone is more metabolically active and responsive to hormonal stimuli, resulting in more rapid bone loss.^55^ We would expect that the gene expression changes would be more pronounced in the rapidly remodeling trabecular bone. Moreover, it is important to recognize that the sample sizes were small, and the gene expression was variable, which is to be expected with biological samples. To address potential issues with small sample sizes and nonnormally disturbed gene expression data, Kruskal–Wallis tests were conducted. Additionally, this study is hypothesis-driven rather than being exploratory in nature, which has led us to opt for a targeted microarray approach. Future studies could use Next-Gen sequencing to provide more comprehensive genomic insights. Finally, the first microarray analysis time point was conducted at 4 wk and missed changes in catabolic gene expression that occur during the initial phase of bone loss. However, it is important to note that our study specifically seeks to provide an advanced understanding of matrix and composition changes that occur in the later stages of osteoporosis^28^^,^^45^ and current diagnosis and treatment occurs at later stages. By shedding light on the molecular mechanisms underlying these later stages, our findings can help bridge the existing knowledge gap and potentially contribute to the development of more effective diagnostic methods and treatment strategies for individuals.

Recent research has established that a deficiency of estrogen after menopause causes a significant decrease in bone volume and changes in the microarchitecture of the trabecular bone in the proximal tibial metaphyses of rats.^25^^,^^28^^,^^45^^,^^53^^,^^56^^,^^57^ Our previous study has shown that bone loss occurs within the first 4 wk after ovariectomy and is characterized by a decrease in trabecular number, whereas in the longer term trabecular, thickness is increased and secondary tissue mineralization occurs.^28^ In the current study, we demonstrated short-term trabecular bone loss occurred within the first 4 wk after ovariectomy corroborating our previous study findings and in longer term estrogen deficiency cortical bone loss occurred. We further employed Raman spectroscopy to measure molecular changes at a microscopic level and to investigate the alterations in matrix quality at different stages of bone loss. After 14 wk of estrogen depletion, OVX animals exhibited more heterogeneous collagen maturity to which extracellular matrix markers COL1A1 and COL1A2 were positively correlated. Interestingly, a study using FTIR to examine the iliac crest of female fragility fracture patients reported an increase of one unit in the heterogeneity of collagen maturity was associated with a 54.9% higher probability of fracture.^24^ In long-term estrogen deficiency, the increase in the heterogeneity of collagen occurs after severe bone loss has subsided. Previous studies have confirmed that collagen and matrix composition can continue to deteriorate.^58-66^ Here, the observed increase in collagen maturity heterogeneity reflects the intricate matrix interactions occurring during prolonged estrogen deficiency.

The current study sheds light on the putative mechanisms by which secondary mineralization and changes in matrix deposition occur. Specifically, in long-term estrogen deficiency, we report increased expression of genes associated with extracellular matrix deposition (COL1A1, COL1A2, OCN). While short-term estrogen deficiency (4 wk) decreased bone matrix deposition and osteoblast differentiation gene expression (ALP, DMP1), we report for the first time that matrix and mineral gene expression (COL1A1, COL1A2, and OCN) increased in the later stage of estrogen deficiency (14 wk postovariectomy) (Figure 4). This may appear contradictory, considering the conventional understanding that the disease primarily involves bone loss. However, recent studies have shed light on changes in tissue properties in postmenopausal osteoporosis.^14-17^^,^^21^^,^^22^^,^^26^^,^^28^^,^^31^^,^^45^^,^^58-70^ Initially, there is a phase of high bone turnover, involving both bone loss and bone formation, followed by a more stable remodeling process.^52^ During prolonged estrogen deficiency, bone resorption and formation persist but occur at a reduced pace.^52^^,^^71^ These results might explain the reported heterogeneity in mineral-to-matrix ratio and collagen maturity reported herewith. Indeed, Lox expression was significantly upregulated 14 wk postovariectomy compared with 4 wk and is known to play a crucial role in the enzymatic crosslinking of collagen in the extracellular matrix.^72^^,^^73^ Specifically, osteoblast differentiation is altered in response to impaired extracellular matrix structure resulting from Lox inhibition with altered expression of COL1A1, and OCN at the mRNA level.^72^ The increased expression of matrix and mineral genes (COL1A1, COL1A2, and OCN) after 14 wk of estrogen deficiency may suggest a secondary reinforcement response, possibly indicating changes in bone structure and increased heterogeneity. This may contribute to material heterogeneity observed in long-term estrogen deficiency and to an increased risk of fractures, as observed in human fracture patients.^24^ In our study OVX animals exhibited more heterogenous mineral, to which extracellular matrix marker, fibronectin, was positively correlated. Fibronectin is a ligand for integrins and enables osteocyte and osteoblast interaction with extracellular matrix proteins and growth factors such as BMPs, impacting bone formation, remodeling, and mineralization processes.

Osteocytes play a crucial role in maintaining bone homeostasis by regulating osteoblastogenesis and osteoclastogenesis. In vitro studies have shown that cells become more sensitive to mechanical stimulation after estrogen depletion and in vivo studies have shown that integrin β3 expression is reduced in osteocytes 4 wk postovariectomy.^32^ Our current study indicates that the expression of genes involved in mechanotransduction or mechanosensation (Vcl, Itga5, Itgb1, Cx43, Esr1, TRPV4, and Adcy6) decreased in 4 wk OVX relative to 4 wk SHAM. Moreover, Vcl, Itgb1, Cx43, Esr1, Axin2, IFT88, and TRPV4 increased in ovariectomized animals at 14 wk compared with week 4 (Figure 4). This suggests a dynamic and time-dependent regulation of these genes in response to ovariectomy. Previous in vitro studies on integrins, particularly the role of integrin αvβ3, revealed the absence of estrogen triggered alterations in mechanotransduction.^32^^,^^74-76^ Our exploration into MLO-Y4 cell morphology, α_v_β_3_ expression, focal adhesion, and mechanotransduction during estrogen withdrawal uncovered a link between estrogen deficiency, defective αvβ3 signaling, and bone loss in postmenopausal osteoporosis.^74^ Our current study further indicates a significant upregulation in mature osteocyte marker PHEX, implicated in osteogenic cell differentiation/bone mineralization, occurred in ovariectomized animals at 14 wk compared with animals at week 4. Interestingly, mineral heterogeneity and cortical bone volume fraction were negatively correlated with PHEX and infraflagellar transport protein, IFT88, implicated in the formation of mechanosensory primary cilia. Primary cilia associated hedgehog (Hh) signaling is an antagonist of osteogenic differentiation by the Wnt signaling pathway.^77^ Thus, increased mineralization might be partially explained by a reduction in Hh signaling. However, in vitro, we reported primary cilia elongation and activation of Hh signaling in response to short-term estrogen deficiency.^76^ Estrogen withdrawal led to cilium elongation, increased Hedgehog and osteoclastogenic signaling, disrupted focal adhesions, and altered actin contractility. The disorganization of αvβ3 integrins played a pivotal role in these processes, contributing to cilium elongation and the activation of osteoclastogenic paracrine signaling. Another noteworthy discovery in our study is that Cx43 was upregulated in ovariectomized rats compared with age-matched controls at wk 14. Given the role of Cx43 in osteocyte gap junctions, this might indicate changes in osteocyte communication. However, in osteoporotic bone, there is decreased connectivity and dendritic disorientation^78^ and osteocytes in estrogen-deficient conditions tend to be smaller, more rounded, and have fewer dendrites. Moreover, estrogen withdrawal in osteocytes attenuates fluid flow-induced intracellular calcium signaling, downstream signaling (PGE_2_, NO), and actin cytoskeleton remodeling, all indicative of altered osteocyte mechanosensitivity.^49^ In this study, the calcium signaling marker TRPV4 was significantly upregulated in ovariectomized rats at week 14 compared with week 4, thus reinforcing these changes in osteocyte communication and signaling over time in estrogen deficiency*.* The Cx43 upregulation also showed a negative correlation with mean mineral density at wk 14. Fluid flow shear stress has been shown to induce the translocation of Cx43 to the membrane surface, and unopposed hemichannels formed by Cx43 serve as a portal for the release of PGE2, a skeletal anabolic agent that can increase bone mass in animals, in response to mechanical strain.^79^ Interestingly, long-term release of PGE2 is significantly reduced in cultured osteoporotic bone cells compared with control.^80^

This study provides novel insights into the temporal nature of gene expression during estrogen deficiency and its implications for postmenopausal osteoporosis treatment. It reveals that although trabecular bone loss occurs within the first 4 wk of estrogen deficiency, catabolic gene expression subsequently abates and increased expression of bone anabolic genes (COL1A1, COL1A2, OCN) occurs in longer term estrogen deficiency. These findings provide mechanistic insight into the secondary compositional changes that occur, which were confirmed here in terms of changes in tissue mineralization, mineral-to-matrix ratio, and collagen maturity. We propose that altered mechanobiological responses in long-term estrogen deficiency may play a role in the observed secondary mineralization. These important findings may inform future interventions that can target bone matrix formation and mechanosensation and thereby address mineral heterogeneity to enhance bone quality and to mitigate bone loss and fracture in postmenopausal osteoporosis.

## Supplementary Material

JBMRPlus_Supplementary_material_ziae098

## Data Availability

The data supporting the findings of this study are available within the manuscript and its supplementary materials. Raw data, including gene expression profiles and histological images, and any other data related to this study are available from the corresponding author on reasonable request.
