# Supplementary material for: Altered extracellular matrix and mechanotransduction gene expression in rat bone tissue following long-term estrogen deficiency
Source: JBMR Plus. 2024 Jul 24;8(9):ziae098. doi: 10.1093/jbmrpl/ziae098 (PMC11347883; doi:10.1093/jbmrpl/ziae098)
Supplement: JBMRPlus_Supplementary_material_ziae098 [file jbmrplus_supplementary_material_ziae098.docx]

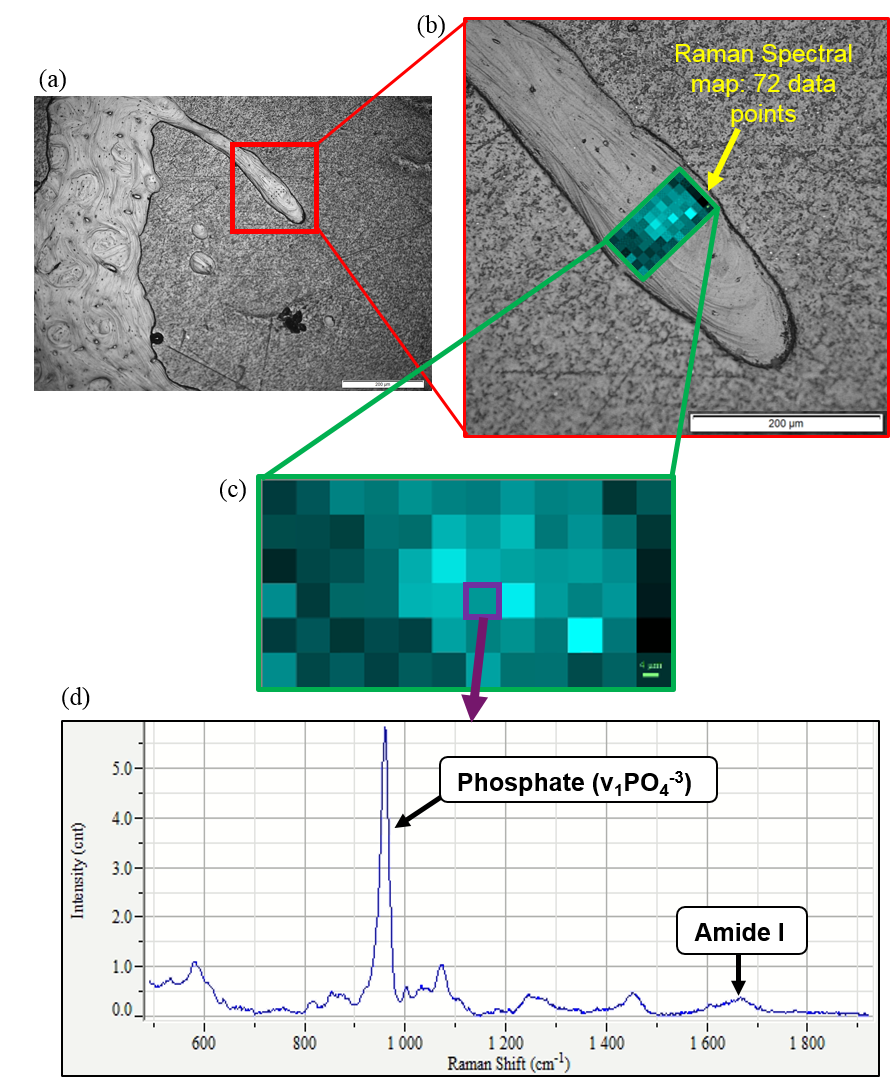


***Figure S1:*** *Schematic illustration of Raman spectroscopy analysis of individual trabeculae of the proximal tibia. (a) Longitudinally sectioned trabecular struts were identified in the sectioned and embedded sample, (b, c) a rectangular Raman spectral map (48µm x 96µm) across the trabecula width (example map shown depicts mineral-to-matrix ratio calculated for each pixel, brightest blue represents the highest value), (d) the Raman intensity and integrated area of the phosphate and amide I peaks were obtained from each spectrum for calculation of matrix composition parameters.*

**
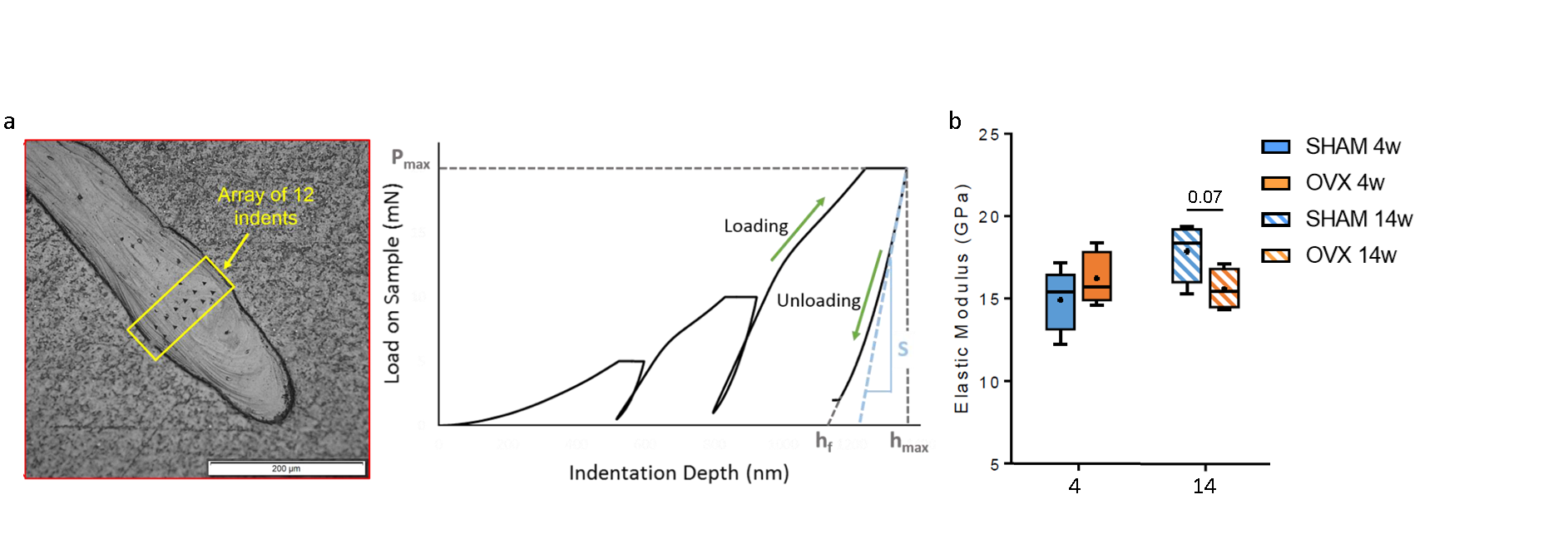
**

***Figure S2:*** *Nanoindentation of individual trabeculae from ovariectomised and age matched control animals, which were sacrificed 4 and 14 weeks after surgery. (a) Representative image of individual trabeculae, which were tested by nanoindentation, schematic of a typical load-indentation depth curve. Pmax = maximum load, S = contact stiffness, hmax = maximum indentation depth, hf = final displacement. (b) Tissue level elastic modulus. Whiskers extend between min and max values of data set, the box extends from 25^th^ to 75^th^ percentiles, horizontal line across the box is the median value and the black dot represents the mean of the data.*


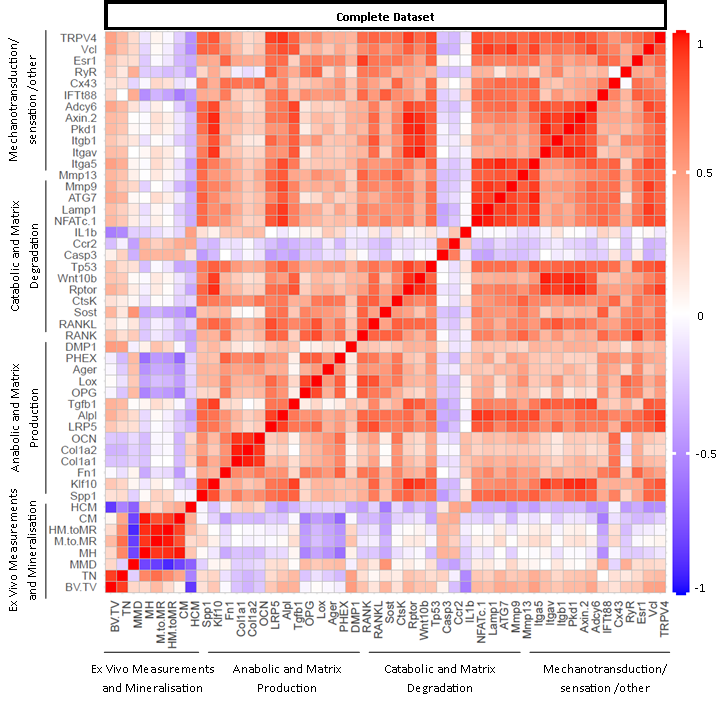


***Figure S3:*** *Correlation matrix comparing trabecular tissue microarchitecture and composition with expression of genes associated with bone anabolism and ECM production, bone catabolism and ECM degradation and mechanotransduction and mechanosensation for the complete dataset (week 4 and week 14 SHAM and OVX groups); dark blue = negative correlation, dark red = positive correlation, white = no correlation.*


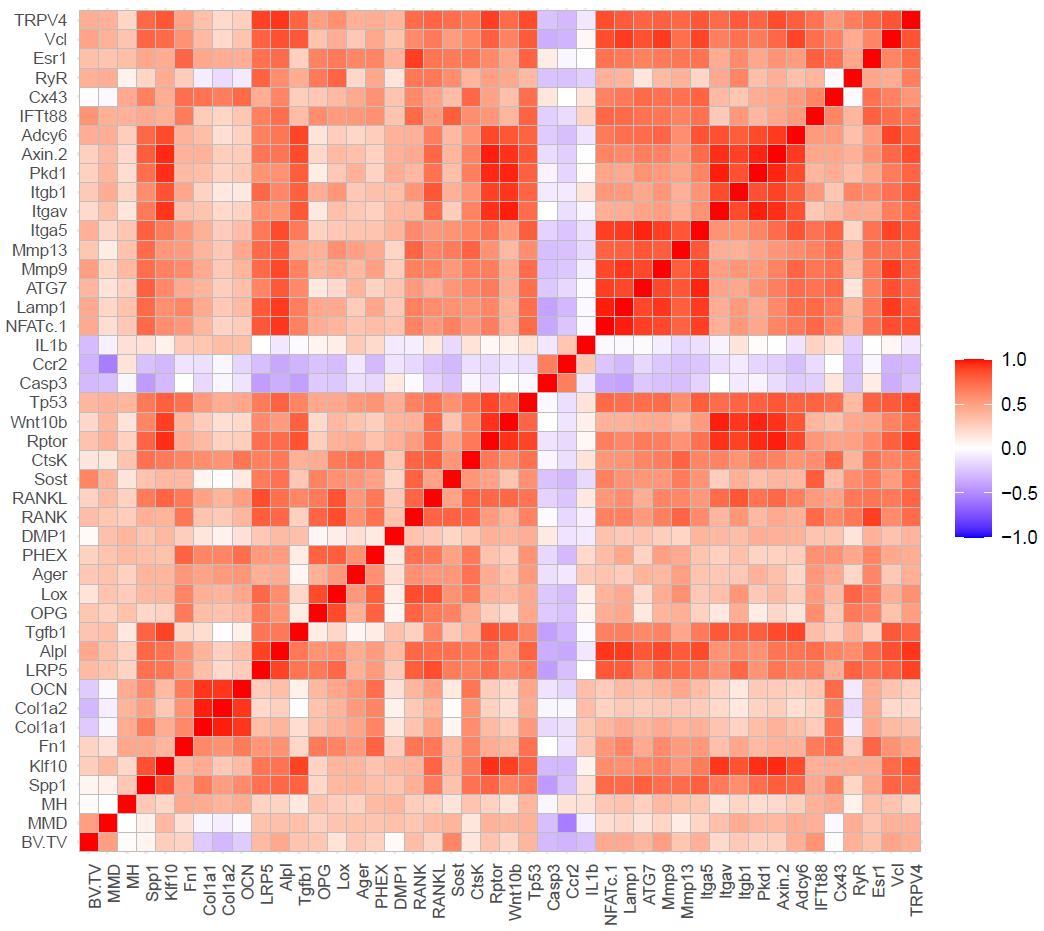


***Figure S4:*** *Correlation matrix comparing cortical tissue microarchitecture and composition with expression of genes associated with bone anabolism and ECM production, bone catabolism and ECM degradation and mechanotransduction and mechanosensation for the complete dataset (week 4 and week 14 SHAM and OVX groups); dark blue = negative correlation, dark red = positive correlation, white = no correlation.*


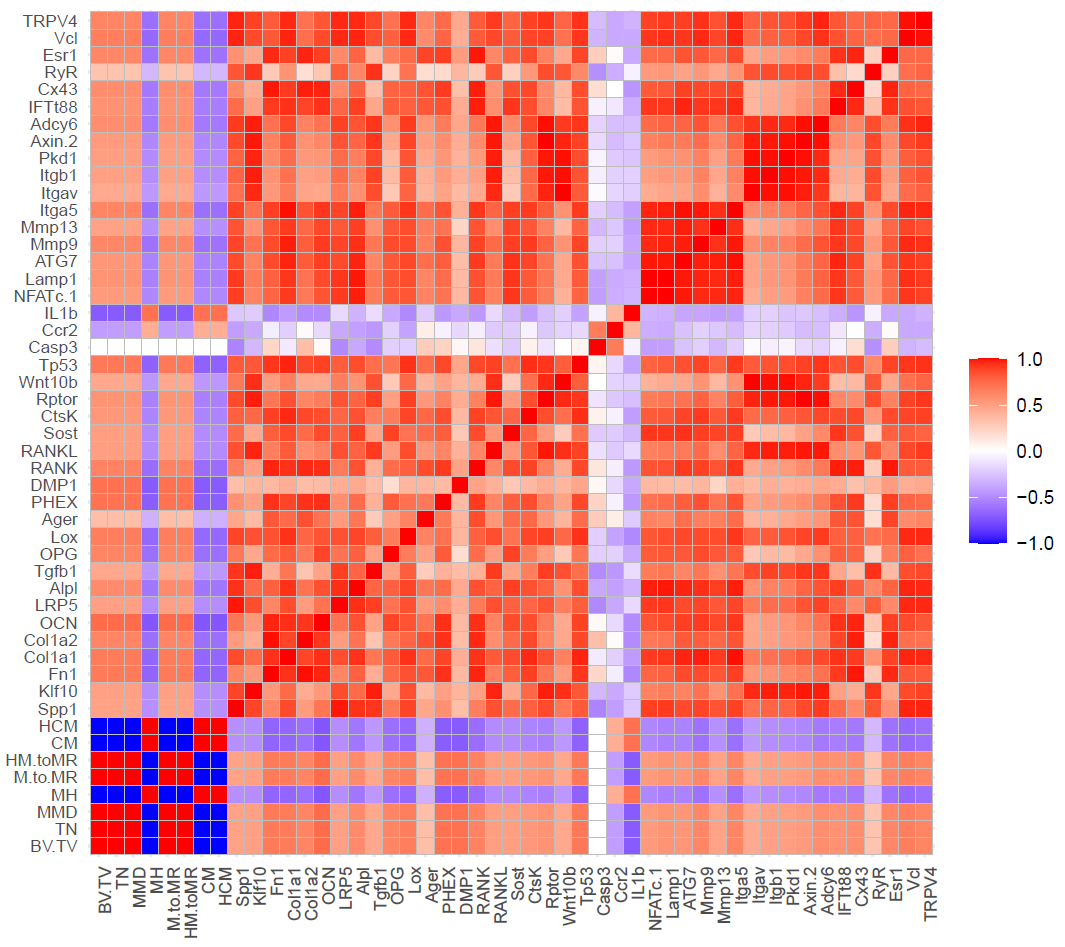


Figure S5: Correlation matrix comparing (a) tissue microarchitecture and composition with (b) expression of genes associated with bone anabolism and ECM production, (c) bone catabolism and ECM degradation and (d) mechanotransduction and mechanosensation for week 4 SHAM and OVX groups only; dark blue = positive correlation, dark red = negative correlation, white = no correlation.


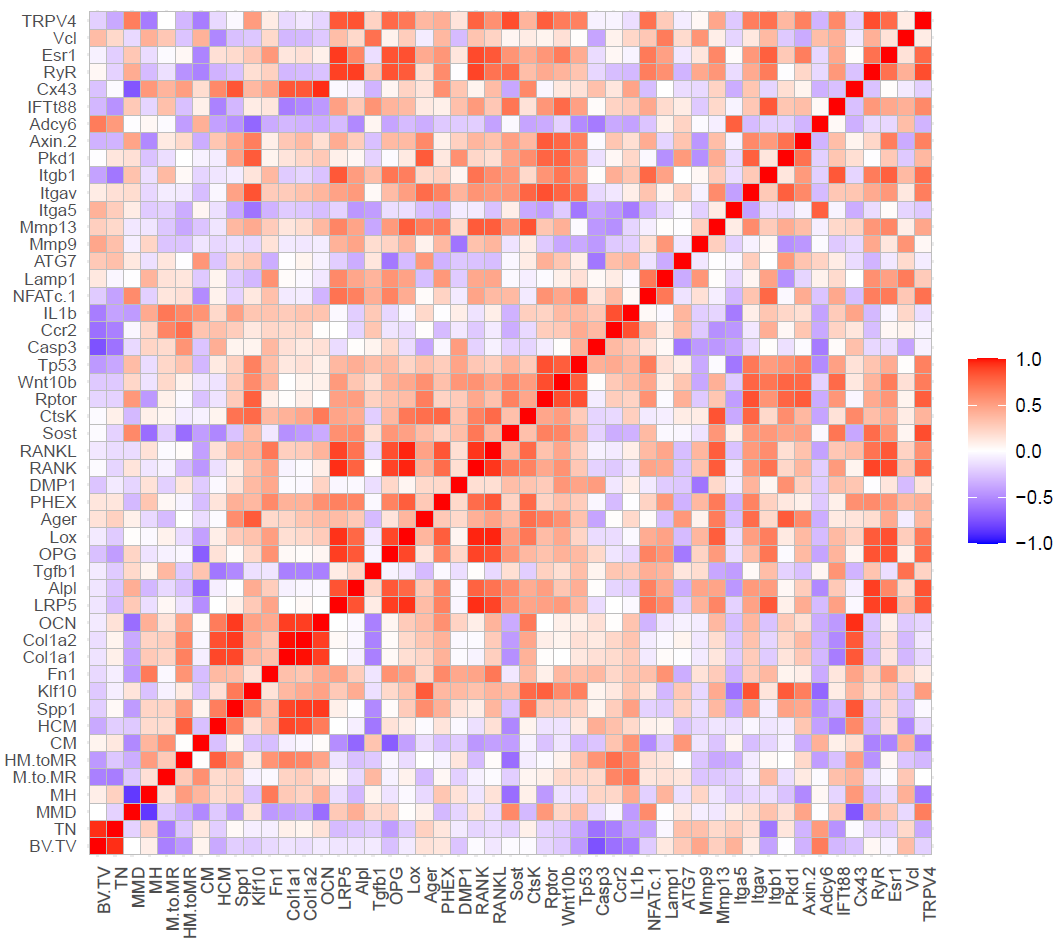


Figure S6: Correlation matrix comparing (a) tissue microarchitecture and composition with (b) expression of genes associated with bone anabolism and ECM production, (c) bone catabolism and ECM degradation and (d) mechanotransduction and mechanosensation for week 14 SHAM and OVX groups only; dark blue = positive correlation, dark red = negative correlation, white = no correlation.


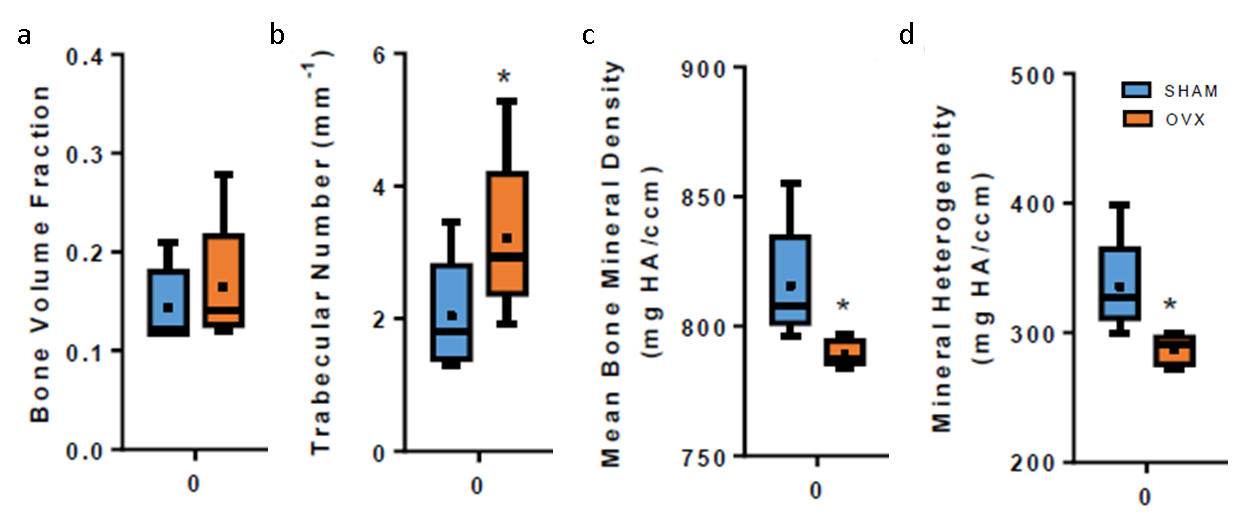


Figure S6: (a-d) Microarchitectural and mineralisation parameters obtained from in vivo micro-CT at baseline (day 0) of the week 14 SHAM and OVX animals from this ex-vivo characterisation study. p<0.05 was considered significant. ‘*’ indicates a significant difference between SHAM and OVX animals.

Table S1: Trabecular and Cortical microarchitecture and mineralisation measurements obtained from high resolution micro-CT analysis of the proximal tibia from SHAM and OVX rats sacrificed 4 or 14 weeks after surgery. Mean ± SD presented for each dataset. P-values presented for SHAM vs OVX comparison at week 4 and week 14 after surgery.

|  |  | **SHAM W4** | **OVX W4** | **Trend & p-value** | | **SHAM W14** | **OVX W14** | **Trend & p-value** | |
| --- | --- | --- | --- | --- | --- | --- | --- | --- | --- |
| **Trabecular** | **Bone Volume Fraction (BV/TV)** | 0.189 ± 0.043 | 0.108 ± 0.026 | **↓** | **0.0004** | 0.148 ± 0.044 | 0.091 ± 0.049 | **ns↓** | 0.08 |
|  | **Mean Mineral Density (mgHA/ccm)** | 1110 ± 25.95 | 1108 ± 27.09 | **ns** | 0.91 | 1135 ± 15.45 | 1111 ± 21.30 | **ns↓** | 0.08 |
|  | **Mineral Heterogeneity (mgHA/ccm)** | 245.9 ± 40.47 | 248.3 ± 28.67 | **ns** | 0.92 | 204.5 ± 12.47 | 216.8 ± 10.34 | **ns** | 0.13 |
| ***Cortical*** | ***Bone Volume Fraction (BV/TV)*** | *0.592 ± 0.04* | *0.575 ± 0.01* | ***ns*** | ***0.5542*** | *0.668 ± 0.01* | *0.572 ± 0.03* | ***↓*** | *0.0204* |
|  | ***Mean Mineral Density (mgHA/ccm)*** | *1151 ± 26.71* | *1103 ± 42.84* | ***ns*** | *0.1612* | *1184 ± 1.69* | *1133 ± 10.05* | ***↓*** | *0.0026* |
|  | ***Mineral Heterogeneity (mgHA/ccm)*** | *251.3 ± 11.87* | *188.6 ± 93.31* | ***ns*** | *0.3297* | *241.5 ± 5.42* | *274.2 ± 1.15* | ***↓*** | *0.0014* |

*ns = not significant*

Table S2: Bone matrix composition parameters obtained from Raman spectroscopy of trabeculae from distinct anatomical regions in the proximal tibia of ovariectomised and age-matched control rats which were sacrificed at 4 and 14 weeks after surgery. Mean ± SD presented for each dataset. P-values presented for SHAM vs OVX comparison at week 4 and week 14 after surgery.

|  | **SHAM W4** | **OVX W4** | **Trend & p-value** | | **SHAM W14** | **OVX W14** | **Trend & p-value** | |
| --- | --- | --- | --- | --- | --- | --- | --- | --- |
| **Heterogeneity of Mineral to Matrix Ratio** | 4.128 ± 1.215 | 3.806 ± 0.928 | **ns** | 0.80 | 2.179 ± 0.117 | 3.192 ± 0.476 | **↑** | 0.03 |
| **Heterogeneity of Collagen Maturity (x10^-3^)** | 4.170 ± 0.687 | 6.260 ± 1.934 | **ns** | 0.22 | 3.746 ± 0.439 | 6.213 ± 1.443 | **↑** | 0.01 |

Table S3: Correlation Table of trabecular ex-vivo microarchitecture and mineralisation data

|  | **BV/TV** | **TN** | **MMD** | **MH** |
| --- | --- | --- | --- | --- |
| **M.to.MR** | ns | r=0.72; p<0.001 | r=-0.83; p<0.001 | r=0.91; p<0.001 |
| **HM.toMR** | ns | r=0.59; p=0.01 | r=-0.91; p<0.001 | r=0.94; p<0.001 |
| **CM** | ns | r=0.46; p=0.04 | r=-0.79; p<0.001 | r=0.98; p<0.001 |
| **HCM** | r=-­0.85; p<0.001 | r=-0.54; p=0.01 | r=-0.69; p=0.001 |  |

*ns = not significant*

*r = correlation magnitude*

*p = p-value*

Table S4: Correlation table of mechanotransduction and mechanosensation gene expression data to bone anabolism and extracellular matrix production and bone catabolism and extracellular matrix degradation gene expression data.

|  | **Itga5** | **Itgav** | **Itgb1** | **Pkd1** | **Axin.2** | **Adcy6** | **IFTt88** | **Cx43** | **RyR** | **Esr1** | **Vcl** | **TRPV4** |
| --- | --- | --- | --- | --- | --- | --- | --- | --- | --- | --- | --- | --- |
| **Spp1** | r=0.77; p<0.001 | r=0.66; p=0.001 | r=0.58; p=0.007 | r=0.71; p<0.001 | r=0.79; p<0.001 | r=0.74; p<0.001 | ns | r=0.64; p=0.002 | ns | r=0.46; p=0.04 | r=0.75; p<0.001 | r=0.75; p<0.001 |
| **Klf10** | r=0.65; p=0.002 | r=0.92; p<0.001 | r=0.83; p<0.001 | r=0.94; p<0.001 | r=0.95; p<0.001 | r=0.86; p<0.001 | ns | ns | ns |  | r=0.74; p<0.001 | r=0.82; p<0.001 |
| **Fn1** | r=0.52; p=0.02 | ns | r=0.46; p=0.04 | ns | ns | ns | r=0.65; p=0.002 | r=0.71; p<0.001 | ns | r=0.75; p<0.001 | r=0.56; p=0.01 | r=0.48; p=0.03 |
| **COL1A1** | ns | ns | ns | ns | ns | ns | ns | r=0.69; p<0.001 | ns | r=0.45; p=0.04 | ns | ns |
| **COL1A2** | ns | ns | ns | ns | ns | ns | ns | r=0.65; p=0.002 | ns | ns | ns | ns |
| **OCN** | ns | ns | ns | ns | ns | ns | ns | r=0.74; p<0.001 | ns | ns | ns | ns |
| **LRP5** | r=0.67; p=0.001 | r=0.57; p=0.01 | r=0.75; p<0.001 | r=0.55; p=0.01 | r=0.69; p=0.001 | r=0.64; p=0.002 | r=0.64; p=0.002 | ns | r=0.76; p<0.001 | r=0.71; p<0.001 | r=0.77; p<0.001 | r=0.89; p<0.001 |
| **Alpl** | r=0.85; p<0.001 | r=0.54; p=0.01 | r=0.60; p=0.004 | r=0.55; p=0.01 | r=0.68; p=0.001 | r=0.69; p=0.001 | r=0.71; p<0.001 | r=0.62; p=0.004 | r=0.58; p=0.007 | r=0.72; p<0.001 | r=0.84; p<0.001 | r=0.92; p<0.001 |
| **Tgfb1** | r=0.66; p=0.001 | r=0.80; p<0.001 | r=0.77; p<0.001 | r=0.79; p<0.001 | r=0.86; p<0.001 | r=0.87; p<0.001 | ns | ns | ns | ns | r=0.80; p<0.001 | r=0.76; p<0.001 |
| **OPG** | ns | ns | ns | ns | ns | ns | r=0.57; p=0.008 | ns | r=0.67; p=0.001 | r=0.63; p=0.003 | ns | r=0.49; p=0.03 |
| **Lox** | ns | ns | r=0.53; p=0.01 | ns | ns | ns | r=0.51; p=0.02 | ns | r=0.75; p<0.001 | r=0.67; p=0.001 | ns | r=0.57; p=0.01 |
| **Ager** | ns | ns | ns | ns | ns | ns | r=0.53; p=0.02 | ns | ns | r=0.60; p=0.005 | ns | ns |
| **PHEX** | ns | ns | ns | ns | ns | ns | r=0.56; p=0.009 | r=0.56; p=0.01 | r=0.44; p=0.05 | r=0.61; p=0.004 | r=0.45; p=0.05 | ns |
| **RANK** | r=0.58; p=0.01 |  | r=0.54; p=0.01 | ns | ns | ns | r=0.74; p<0.001 | r=0.59; p=0.005 | r=0.67; p=0.001 | r=0.90; p<0.001 | r=0.58; p=0.006 | r=0.73; p<0.001 |
| **RANKL** | r=0.53; p=0.02 | r=0.73; p<0.001 | r=0.82; p<0.001 | r=0.69; p=0.001 | r=0.74; p<0.001 | r=0.64; p=0.002 | r=0.52; p=0.02 | r=0.47; p=0.03 | r=0.68; p=0.001 | r=0.69; p=0.001 | r=0.66; p=0.001 | r=0.76; p<0.001 |
| **Sost** | r=0.55; p=0.01 | ns | ns | ns | ns | ns | r=0.79; p<0.001 | ns | r=0.58; p=0.01 | r=0.66; p=0.001 | r=0.50; p=0.02 | r=0.72; p<0.001 |
| **CtsK** | r=0.62; p=0.003 | r=0.62; p=0.003 | r=0.54; p=0.01 | r=0.65; p=0.002 | r=0.63; p=0.003 | r=0.54; p=0.01 | r=0.57; p=0.01 | r=0.75; p<0.001 | ns | r=0.68; p=0.001 | r=0.62; p=0.003 | r=0.69; p=0.001 |
| **Rptor** | r=0.71; p<0.001 | r=0.93; p<0.001 | r=0.89; p<0.001 | r=0.95; p<0.001 | r=0.96; p<0.001 | r=0.86; p<0.001 | r=0.54; p=0.01 | r=0.47; p=0.03 | r=0.48; p=0.03 | r=0.59; p=0.005 | r=0.77; p<0.001 | r=0.89; p<0.001 |
| **Wnt10b** | r=0.51; p=0.02 | r=0.96; p<0.001 | r=0.92; p<0.001 | r=0.96; p<0.001 | r=0.93; p<0.001 | r=0.81; p<0.001 | ns | ns | r=0.44; p=0.05 | r=0.47; p=0.04 | r=0.63; p=0.003 | r=0.73; p<0.001 |
| **TP53** | r=0.78; p<0.001 | r=0.73; p<0.001 | r=0.76; p<0.001 | r=0.78; p<0.001 | r=0.82; p<0.001 | r=0.77; p<0.001 | r=0.76; p<0.001 | r=0.71; p<0.001 | ns | r=0.77; p<0.001 | r=0.79; p<0.001 | r=0.85; p<0.001 |
| **NFATc.1** | r=0.90; p<0.001 | ns | r=0.52; p=0.02 | r=0.45; p=0.04 | r=0.61; p=0.004 | r=0.67; p=0.001 | r=0.75; p<0.001 | r=0.63; p=0.003 | ns | r=0.69; p=0.001 | r=0.84; p<0.001 | r=0.85; p<0.001 |
| **Lamp1** | r=0.90; p<0.001 | ns | r=0.52; p=0.02 | ns | r=0.59; p=0.005 | r=0.71; p<0.001 | r=0.73; p<0.001 | r=0.68; p=0.001 | ns | r=0.68; p=0.001 | r=0.90; p<0.001 | r=0.80; p<0.001 |
| **ATG7** | r=0.95; p<0.001 | r=0.46; p=0.04 | r=0.44; p=0.05 | r=0.55; p=0.01 | r=0.64; p=0.002 | r=0.72; p<0.001 | r=0.69; p=0.001 | r=0.73; p<0.001 | ns | r=0.63; p=0.003 | r=0.84; p<0.001 | r=0.76; p<0.001 |
| **Mmp9** | r=0.90; p<0.001 | r=0.50; p=0.02 | r=0.53; p=0.01 | r=0.51; p=0.02 | r=0.62; p=0.003 | r=0.76; p<0.001 | r=0.68; p=0.001 | r=0.70; p=0.001 | ns | r=0.67; p=0.001 | r=0.90; p<0.001 | r=0.77; p<0.001 |
| **Mmp13** | r=0.81; p<0.001 | ns | ns | r=0.47; p=0.04 | r=0.53; p=0.01 | r=0.57; p=0.01 | r=0.63; p=0.003 | r=0.70; p<0.001 | ns | r=0.68; p=0.001 | r=0.72; p<0.001 | r=0.74; p<0.001 |

*ns = not significant*

*r = correlation magnitude*

*p = p-value*

Table S5. Correlation Table of trabecular ex-vivo data to gene expression data at 4 weeks only

| Gene | BV/TV | TN | MH |
| --- | --- | --- | --- |
| Spp1 | ns | r=0.89; p=0.0004 | ns |
| Klf10 | ns | r=0.80; p=0.004 | ns |
| Fn1 | r=0.67; p=0.03 | ns | ns |
| COL1A1 | ns | r=0.75; p=0.01 | ns |
| OCN | r=0.69; p=0.02 | ns | ns |
| LRP5 | ns | r=0.88; p=0.0006 | ns |
| Alpl | ns | r=0.87; p=0.001 | ns |
| Tgfb1 | ns | r=0.85; p=0.001 | ns |
| Lox | r=0.72; p=0.02 | r=0.81; p=0.004 | ns |
| RANKL | ns | r=0.73; p=0.02 | r=0.67; p=0.03 |
| Sost | ns | r=0.66; p=0.04 | ns |
| Rptor | ns | r=0.75; p=0.01 | ns |
| Wnt10b | ns | ns | r=0.67; p=0.03 |
| TP53 | r=0.65; p=0.04 | r=0.70; p=0.02 | ns |
| CCR2 | ns | r=-0.67; p=0.03 | ns |
| IL1b | r=-0.79; p=0.01 | ns | ns |
| NFATc.1 | ns | r=0.81; p=0.004 | ns |
| Lamp1 | ns | r=0.82; p=0.003 | ns |
| ATG7 | ns | r=0.75; p=0.01 | ns |
| Mmp9 | ns | r=0.78; p=0.008 | ns |
| Mmp13 | ns | r=0.69; p=0.02 | ns |
| Itga5 | ns | r=0.79; p=0.006 | ns |
| Itgav | ns | ns | r=0.67; p=0.03 |
| Itgb1 | ns | r=0.67; p=0.03 | ns |
| Pkd1 | ns | r=0.67; p=0.03 | r=0.67; p=0.03 |
| Axin.2 | ns | r=0.76; p=0.01 | r=0.65; p=0.04 |
| Adcy6 | ns | r=0.81; p=0.004 | ns |
| RyR | ns | r=0.73; p=0.02 | ns |
| Vcl | ns | r=0.87; p=0.0008 | ns |
| TRPV4 | ns | r=0.87; p=0.001 | ns |

*ns = not significant*

*r = correlation magnitude*

*p = p-value*

Table S6. Correlation Table of trabecular ex-vivo data to gene expression data at 14 weeks only

| Gene | BV/TV | MMD | HM.toMR | CM | HCM |
| --- | --- | --- | --- | --- | --- |
| COL1A1 | ns | ns | ns | ns | r=0.87; p=0.001 |
| COL1A2 | ns | ns | ns | ns | r=0.84; p=0.002 |
| OPG | ns | ns | ns | r=-0.70; p=0.02 | ns |
| Casp3 | r=-0.74; p=0.01 | ns | ns | ns | ns |
| CCR2 | ns | ns | r=0.71; p=0.02 | ns | ns |
| Cx43 | ns | r=-0.72; p=0.02 | ns | ns | ns |

*ns = not significant*

*r = correlation magnitude*

*p = p-value*
